# Late Occurrence of Transient Advanced Second Degree Atrioventricular Block after Successful Transcatheter Cryoablation of Atrioventricular Nodal Reentry Tachycardia

**DOI:** 10.1155/2012/752956

**Published:** 2012-10-14

**Authors:** Maria Malaya C. Dorotan-Guevara, Michael S. Crapanzano, Christopher S. Snyder

**Affiliations:** ^1^Department of Pediatric Cardiology, University of Florida School of Medicine, Gainsville, FL 32611, USA; ^2^Pediatric Cardiology Associates, Baton Rouge, LA 70808, USA; ^3^Department of Pediatric Cardiology, Rainbow Babies and Children's Hospital, Case Western Reserve School of Medicine, 11100 Euclid Avenue, MS RBC 6011, Cleveland, OH 44106, USA

## Abstract

Late occurrence of atrioventricular nodal block is an extremely rare occurrence after radiofrequency catheter modification of the slow pathway and has yet to be reported after cryoablation. We report a case of late transient advanced second degree atrioventriuclar block after cryomodification of the slow pathway.

## 1. Introduction

Atrioventricular nodal reentrant tachycardia is a common form of supraventricular tachycardia in children accounting for 13% to 23% of tachycardia [1]. Radiofrequency catheter ablation (RFCA) and cryoablation have evolved into safe and effective treatments of such arrhythmias, in lieu of life-long drug therapy [2, 3]. As with every procedure, potential risks exist, such as developing atrioventricular (AV) block acutely during delivery of RF energy in the septal region [4]. Nonetheless, there is little data on late AV block, which is defined as block developing 24 hours after RFCA or cryoablation [4]. Thus, we report a case of a 16-year-old male who underwent successful combined ablation (RFCA and cryoablation) for AV nodal reentry tachycardia who developed transient advanced second degree AV block 24 hours post successful ablation with complete resolution 10 weeks post-procedure.

## 2. Discussion

M. F. is an otherwise healthy, 16-year-old male who had episodes of supraventricular tachycardia while participating in high endurance athletics. He was referred to our institution for an electrophysiology study and ablation. A four-catheter electrophysiology study was performed under general anesthesia. Preablation baseline intervals show a cycle length of 781 milliseconds (msc), PR interval of 120 msc; QRS was 76 msc, Atrium (A) to His (H) of 49 msc and H to Ventricle (V) of 40 msc and QT of 377 msc. Normal conduction was established with an A pacing Wenckebach cycle length of 260 msc and V pacing Wenckebach cycle length of 470 msc. During a single atrial extra stimulus protocol at 600/290 there was an A-H jump and tachycardia was induced. His supraventricular tachycardia cycle length was 310 msc with an H-A-V tracing noted on the His catheter diagnostic for AV node reentry.

Mapping of the slow pathway was performed utilizing the NAVIX 3D mapping system, GE fluoroscopy, and the Prucka system to identify ideal electrograms (M shaped atrial electrogram and 1 : 3 A to V ratio). Mapping was initially performed to tag the His bundle and coronary sinus then once the ideal electrocardiograms were located, three (3) RF lesions were placed in the low mid septal region with power of 50 watts, maximum temperature 60°C, and impedances ranging from 93–110 ohms. Each lesion resulted in accelerated junctional rhythm with 1 : 1 junctional : atrial conduction.

After RFCA, post-ablation testing reinduced the patient's AVN reentry. Due to the ineffectiveness of RFCA and the need to approach his AV node, the decision was made to change to the cyoablation system (CryoCath, Medtronic, St. Paul Minn). The cryocatheter, a Freezor Xtra 3. (227F3), 6 millimeter tip catheter was utilized to place three, four minute freeze—30 second thaw—4 minute freeze cryoablation lesions. The lowest temperature achieved was −76 degrees, in the mid-slow pathway position. The patient was in sinus rhythm throughout the lesions with no prolongation of PR, AH, or HV intervals were noted. After cryoablation, A pacing and atrial extra stimulus revealed complete modification of slow pathway.

**Figure 1:**
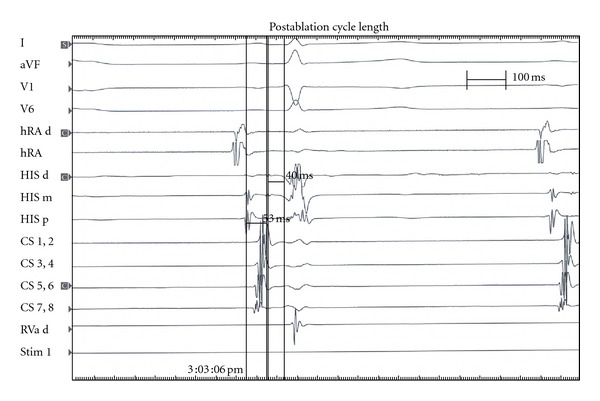


**Figure 2:**
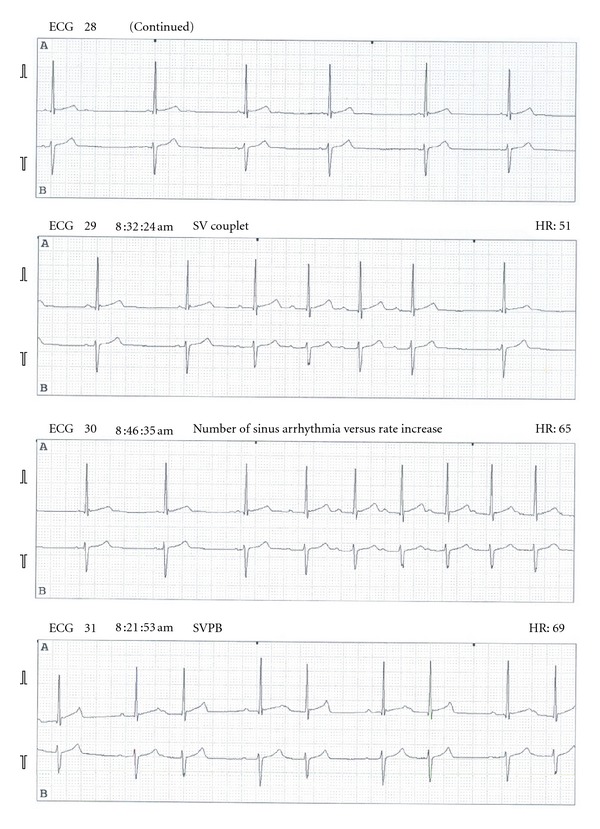
Holter monitor with Wenckebach.

Thirty minutes later, post-ablation testing revealed sinus rhythm with a PR interval of 120 msc, an AH of 53 msc, and an HV of 40 msc (Figure 1). A pacing revealed his Wenckebach cycle length at 270 msc, VA Wenckebach at 210 msc during V pacing. Single atrial extra stimulus found his AV node ERP was 600/250 without an A-H jump or reentry beats. After determining that the patient had no residual slow pathway conduction and no reentry beats, the study was concluded without complications. An electrocardiogram was performed five hours post-RF which revealed normal sinus rhythm. He was discharged home without problems. A 24 hour Holter monitor was placed just prior to discharge that showed sinus rhythm with a peak heart rate of 140 beats per minute for the first nineteen hours then episodes of second degree Type 1 AV block were noted (Figure 2).

**Figure 3:**
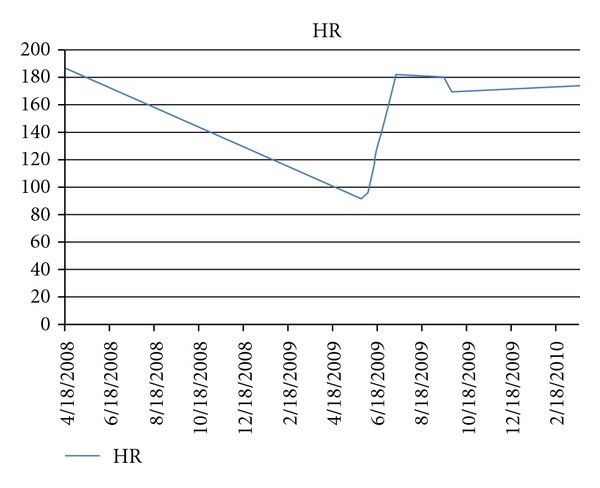
Peak heart rate response to exercise pre- and post-ablation.

The following day, the patient complained of dizziness and chest pain; and was advised to go to his local pediatric cardiologist's office to be evaluated. During his evaluation, he was found to have advanced second degree AV block with resting heart rate of 50 beats per minute. Additional ancillary testing performed were normal, which included an echocardiogram, a chest radiograph, and cardiac enzymes. The patient was admitted to the hospital initially for observation as he was eventually discharged 12 days later. While hospitalized he received steroid therapy, Decadron 4 mg IV daily followed by an oral prednisone wean, to decrease edema and inflammation in the involved area. Upon discharge, his electrocardiogram showed normal sinus rhythm. During his outpatient follow up, serial exercise stress testing utilizing standard Bruce protocol was performed with linear improvement of his Wenckebach rate from a HR of 92 beats per minute to his preprocedure maximum of 182 beats per minute at 10 weeks post procedure (Figure 3).

Atrioventricular block is widely recognized as a major potential complication of RFCA, with the probable need for long-term pacing especially among pediatric patients [5–7]. The Pediatric RFCA registry reports an RFCA associated AV block incidence of 0.88% (1991–1995), 0.56% (1996–1999), and 1.2% (1999–2003) [6]. The frequency is related to the ablation anatomic site: 2.7% anteroseptal, 10.4% midseptal, and 1% right posteroseptal sites; 1.6% for AVNRT [5].

Although there have been reports on adults with late occurrence of AV block following RFCA, its pathogenesis remains to be unclear. Fenelon et al. [8] described a relation between the development of late complete AV block (*P* > 0.0001) and transient AV block during RFCA for AVNRT. A total of 4 patients (all ages > 47 years) developed complete AV block at variable time periods post-ablation (20 hours to 1 month) after ablation with all requiring permanent pacemaker placement (follow up 1–23 months). In addition, a 13-year-old was found to have second degree AV block (Mobitz Type 1) which progressed to 2 : 1 block during exercise testing 3 months after ablation [8]. The mechanism proposed by the authors was related to delayed effects of radiofrequency energy on well demarcated areas of coagulative necrosis surrounded by an inflammatory cell infiltrate and hemorrhage. The tissue surrounding this area may then recover completely, partially, or progress to cell death and posterior fibrosis. It may be possible then that the nontargeted pathway or the compact node is located inside this described inflammatory area [8]. Another possibility involves the microcirculatory damage to the tissue surrounding the central necrotic areas that can result in expansion of the lesion over time [8].

Pelargonio et al. [4] reported occurrence of late heart block in 2 out of 418 (0.5%) of patients with AV node reentry and 1 in 54 (2%) of patients with posteroseptal accessory pathway. All patients were females and age > 27 years with block developing on the second day (2) and third day (1) after the procedure. None of patients had transient AV block, worsening of AV nodal or His-Purkinje conduction after RF energy delivery or at the end of the procedure. One patient received a dual chamber pacemaker while the other 2 were clinically followed. The symptoms and correlating tracings suggestive of AV block resolved 8–16 days after RFCA, with no reported recurrence upon follow up (2 months–1 year). It was postulated that the late, self-terminating heart block was caused by edema in the septal region due to numerous high energy applications [4].

The pediatric experience with regards to this late after effect is even less well explored. The pediatric radiofrequency ablation registry (1991–1994) [5] reported 2 cases of second degree AV block that occurred 24 hours and 2 months after the procedure. The first patient was acutely managed with pacing and steroids while the other was observed with no intervention. Both patients remained in second degree AV block upon follow up (1, 10 months).

Transient AV block associated with cryoablation therapy has been reported to occur between 7–20% of patients [9]. This is probably due to expansion of the lesion created during cryoablation. In these patients, there was note of increased PR interval at the end of the procedure and required hours to days to recover. The only predictor of an increased risk of AV block was the application of cryoenergy at the midseptal region. Although this may be the case, there is no report of an AV block that occurred late following the procedure such as in this patient's case. In this patient, the post-procedure Holter monitor showed that he developed first degree AV block approximately 20 hours into the Holter, which was placed just prior to his discharge from the hospital. He then progressed to symptomatic advanced second degree AV block. There were no factors that we can identify that may have led to such an event. Although further investigation may be warranted, the postulated theory of extending edema and necrosis both due to RF and cryoenergy seem to be a plausible explanation for this rare occurrence. Furthermore, while there have been reported cases of late AV block being permanent, this patient's case illustrates the possibility of resolution after 2-3 weeks, eliminating the need for a permanent pacemaker placement.

## 3. Conclusion

This case report demonstrates the occurrence of late transient advanced second degree atrioventricular (AV) block after a combined ablation procedure (radiofrequency and cryoablation) in a pediatric patient. The cause of this unusual phenomenon is most likely due to extension of inflammation or edema around the septal area. The important point is that the patient's normal atrioventricular conduction returned after watchful waiting.
